# “As long as they eat”? Therapist experiences, dilemmas and identity negotiations of Maudsley and family-based therapy for anorexia nervosa

**DOI:** 10.1186/s40337-019-0255-1

**Published:** 2019-08-01

**Authors:** Jessica Aradas, Diana Sales, Paul Rhodes, Janet Conti

**Affiliations:** 1 University of Western Sydney, Locked Bag 1797, Penrith, 2751 Australia; 2 University of Sydney, Penrith, Australia

**Keywords:** Anorexia nervosa, Maudsley, Family-based therapy, Evidence-based practice, Therapist, Identity

## Abstract

**Background:**

Maudsley Family Therapy and its manualised version Family-Based Therapy for Anorexia Nervosa (FBT-AN) have accrued the most significant research evidence-base for the treatment of adolescent Anorexia Nervosa (AN). A tradition of seeking augmentations for this treatment has also been established to enhance efficacy. There exists, however, a gap in the uptake of this form of manualised treatment into the “real world” of clinicians who work with adolescent AN.

**Aims:**

This research study investigated the key experiences and identity negotiations of a group of nine Australian clinicians who were interested in contributing to research into ways that Maudsley and FBT-AN might be improved.

**Methods:**

Nine clinicians, who at the time of the interview practised or had previously practised, FBT-AN participated in a semi-structured interview. A critical discursive analysis of interview transcripts generated a thematic map of these therapists’ experiences and identity negotiations in their practice of FBT-AN.

**Results:**

These therapists experienced the structure of FBT-AN as both a secure map for therapy, yet also constraining at times, in their work with adolescents and their families. Additionally, their professional identities were both invested and troubled by the identity position of themselves as evidence-based practitioners, particularly where evidence-based practice (EBP) meant strict fidelity to the manual and restrained them from tailoring a broader range of therapeutic interventions to an individual adolescent and their family. Within their narratives, these therapists refashioned alternative identity positions around what it meant to be an evidence-based practitioner through listening to and drawing on their clinical expertise of what works in therapeutic practice with an individual adolescent and their family.

**Conclusions:**

These therapists narratives highlight the power of the dominant discourse of EBP that works to privilege the research evidence over other forms of evidence that include clinician expertise and client preferences. The dilemmas faced by these therapists questioned not only the strict application of FBT-AN for adolescent AN across diverse therapeutic contexts, but also the effects of supervisory practices that paralleled this strict fidelity to the model. Further research is needed into therapeutic interventions and supervisory practices that give greater scope for clinicians to draw on their expertise in the flexible tailoring of treatments to the unique needs and preferences of the individual adolescent and their family.

**Electronic supplementary material:**

The online version of this article (10.1186/s40337-019-0255-1) contains supplementary material, which is available to authorized users.

## Plain English summary

FBT-AN is currently the most prominent approach to the treatment of AN in young people and is supported by a range of empirical studies. Many studies have also been conducted on augmentations to the approach, with care taken not to contravene its core principles. While there is evidence that this approach works for some families, little is known about how clinicians negotiate their identities as both evidence-based clinicians and therapists who rely on their own clinical expertise and values. This study demonstrates that clinicians can feel secure in the knowledge that the model they are using is supported by research, but also troubled when strict adherence contradicts their own judgements about how to best support young people and families.

## Background

Maudsley Family Therapy [1] and its subsequent manualised version Family-Based Therapy for Anorexia Nervosa^1^ [2, 3] (FBT-AN), are considered to be one of the few efficacious treatments for adolescent Anorexia Nervosa (AN) [4–7]. The core principles of the therapy include: externalisation of the illness, positioning of families as the best resource to fight the AN and postponing individual and family issues until weight restoration has been achieved. Therapists are asked to “insist that the parents remain relentless until they are convinced that the patient clearly understands that she will not be able to return to anorexic behaviour while a part of the parents’ household” ([3], p., 126). A substantive body of augmentations to this approach have also been developed and studied, including integration with inpatient and outpatient programs, multiple family therapy, and the inclusion of exposure techniques and cognitive-behavioural therapy for perfectionism [8]. Augmentations have been designed so not to alter the core principles of the original model and there is limited guidance as to how clinicians might decide on which enhancement to use for which situations [8].

The research evidence for FBT-AN has largely been in the form of randomized controlled trials (RCT’s) [6, 7, 9, 10] and systematic reviews [11, 12]. Not including those who dropout from treatment, these family interventions have been found to have good long-term outcomes (including in establishing weight gain and improved eating disorder (ED) symptomatology) for approximately half of medically stable adolescents with a shorter duration of AN and aged between 12 and 18 years [13–15]. More specifically, 40% of adolescents are remitted after FBT-AN (i.e. meeting “high bar” recovery definitions based on symptom remission), however, clinical improvement has been found in around double this number ([16], p., 482). Furthermore, a recent randomised multi-centre trial has found good or intermediate outcomes (on the Morgan-Russell scales) in 60% of adolescents treated with single family FBT and 75% treated with multi-family therapy FBT [17]. On the other hand, when psychological distress has been measured more comprehensively in addition to ED symptom improvement in one RCT, around 40% of the adolescents reported (either themselves or by their parents) at 4 years post FBT-AN to be experiencing “considerable on-going psychological and psychiatric difficulty” ([18], p., 672)

A recent systematic review [11] has highlighted the need for future research into the implementation of FBT-AN into clinical practice in the context of research that has identified barriers to its translation. The research to date has also lacked (1) Comparison with individual psychological interventions; and (2) Usable outcome data of general and family functioning post AN treatment [19]. Furthermore, in the proliferation of research that has augmented FBT-AN with other interventions, only two out of 26 studies have received a strong quality rating at systematic review [8].

Manualised treatments play an important role in standardising and disseminating empirically-supported therapies (EST’s) that may be applied more broadly into clinical contexts [20]. However, Sackett and colleagues' model of evidence-based practice (EBP) constitites three arms that, in addition to the resesarch evidence, also includes clinician expertise and client preference and values [21]. There contines to be limited guidance on, and continued debate about, the extent by which therapists have scope to draw on their clinical expertise to tailor FBT-AN to an individual adolescent, their family and within a potentially complex social environment [22, 23]; particularly when this expertise takes them away from fidelity to the FBT-AN manual. Discourse analysts believe that “facts” and “evidence” are most heatedly drawn upon when there is a sensitive issue at stake [24]. So where and how do ED clinicians position themselves in this debate as they seek to provide manualised FBT-AN for adolescent AN?

Qualitative research provides scope for a comprehensive and nuanced understanding of patient and clinician experiences, therefore elucidating the components of EBP that are less accessible by RCT’s and manualised treatments [25]. Qualitative research into clinicians’ experiences of FBT-AN has focused on factors that have increased or decreased therapists’ willingness to practise FBT-AN [26] and some of the ways that therapists adapt FBT-AN for older adolescents to prioritize the adolescent and family needs [27]. While this research is helpful in exploring some of the ways therapists navigate the research-practice gap [28], it tends to be unidirectional in focus on processes by which clinicians make the decision whether or not to practice or adapt the model. What is less understood are questions such as how a clinician’s professional identity (e.g. experiences, values) shapes their uptake or non-uptake of FBT-AN, how they negotiate a sense of professional identity (or who they understand themselves to be as therapists) when drawing on FBT-AN and how they navigate working within and outside the manualised FBT-AN model. These questions are significant, given that there are a number of factors that may increase the rates of burnout for therapists who work with EDs [29] and there is need to more richly understand how ED therapists negotiate and sustain their professional identities in their work with adolescents and their families.

## Methods

### Participants

Nine Australian clinicians who self-identified themselves as a practitioner (either past or present) in FBT-AN participated in this study. This sample was recruited through two research advertisements in 2016 and 2017 distributed through Australian associations relevant to the practice of psychology or within the ED field. The research advertisement invited clinicians who had worked as FBT-AN therapists to talk about their experiences and to generate constructive feedback through asking them: “How we can improve Maudsley Family Therapy for adolescent anorexia?”

The final sample consisted of seven female and two male therapists: five clinical psychologists, two psychologists, one psychotherapist, and one dietitian. Their years of working within ED’s ranged between 5 and 23 years, and they identified themselves as FBT-AN practitioners for between 1 and 10 years. They had all attended Maudsley and/or FBT-AN training and received supervision by experienced FBT-AN practitioners in Australia. For reasons of confidentiality, this demographic information has not been linked to individual participant narratives.

At the time of the interviews, three clinicians indicated they had discontinued both working within FBT-AN and with adolescent AN, although continued to work with adult EDs (Joy, Gloria and Glenda). The remaining six therapists identified as continuing to use some form of FBT-AN to treat adolescent AN. Table 1 summarises how these therapists reported varying their work from the FBT-AN manual [2, 3].

**Table 1:** Therapists’ reported utilisation of specific FBT-AN manual protocols and use of other therapeutic modalities

Therapists	Always weigh every session?	Always postpone all issues till weight restoration?	Always conduct family meal?	Always externalize “AN”?	Use other models for treating EDs?
Marmot	YES	YES	YES	YES	YES
Josh	YES	YES	NO	YES	YES
Francesca	YES	YES	YES	YES	YES
Anja	YES	NO	NO	YES	YES
Sebastian	NO	NO	NO	YES	YES
Paige	YES	NO	YES	YES	YES
Glenda^a^	YES	NO	YES	YES	YES
Gloria^a^	YES	NO	NO	YES	YES
Joy^a^	YES	NO	YES	YES	YES

^a^No longer practising FBT-AN at time of interview

### Procedure and materials

The research study was approved by the University of Western Sydney Human Research Ethics Committee. On consenting for this research study, participants completed a brief demographic and clinical background questionnaire (Additional file 1).

Data was generated through individual semi-structured interviews. The interview questions (Additional file 2) were drawn from the therapeutic paradigm of narrative therapy [30] and were interested both in the way clinician’s practised FBT-AN as well as the ways they ascribed meaning and negotiated their identities in the context of these experiences.

Two researchers (DS and JA) conducted the 45–60 min individual interviews with participants either in person or over the phone between May 2016 and June 2017. The audio-recordings were then transcribed verbatim using light transcription [31], including the use of pauses, in order to better capture shifts in both language and flow. The transcripts were identifiable only by the participant’s chosen pseudonym. The clinicians were also invited to read through their transcripts to remove further potentially identifying information for reasons of their confidentiality.

### Analysis

Analysis aimed to generate understandings of therapists’ experiences, perspectives and the positioning of their work as FBT-AN practitioners, the language forms that they used to ascribe meaning to these experiences, and some of the ways that they negotiated and preserved their identities within these contexts. Initially, open and focused coding [32] was conducted by two researchers (JA and DS) line-by-line using the qualitative research software program Nvivo 11**©** [33], with the intention of generating multiple perspectives [34] from which themes were constructed. This was followed by memo-writing, conducted by the researchers (JA and DS) and with a group of qualitative researchers (including JC and PR), that analysed the data through a critical discursive paradigm [35, 36]. This included the identification of some of the discursive resources used by participants to piece together their narratives and negotiate personal meanings and social identities. These discursive resources included first, interpretative repertoires or culturally inherited patterns of speech (or small “d” discourse) that the participants used to piece together a range of positions and arguments [36, 37]; and second, positioning in discourse that constitutes the diverse locations within which the participants were both positioned and positioned themselves in discourse. These discourse positionings provided a platform from which the therapists constructed and negotiated a sense of professional identity within a social context [38]. Analysis traced how these therapists’ accounts of their experiences were continually (re)constructed, (re)negotiated, and (re)formed through argument, addressing ideological dilemmas and rhetoric [37, 39].

The clinician participants were invited to read a draft of this paper, in particular to member-check the validity of the analysis of their transcripts in interpreting their experiences.

## Results

**Fig. 1:**
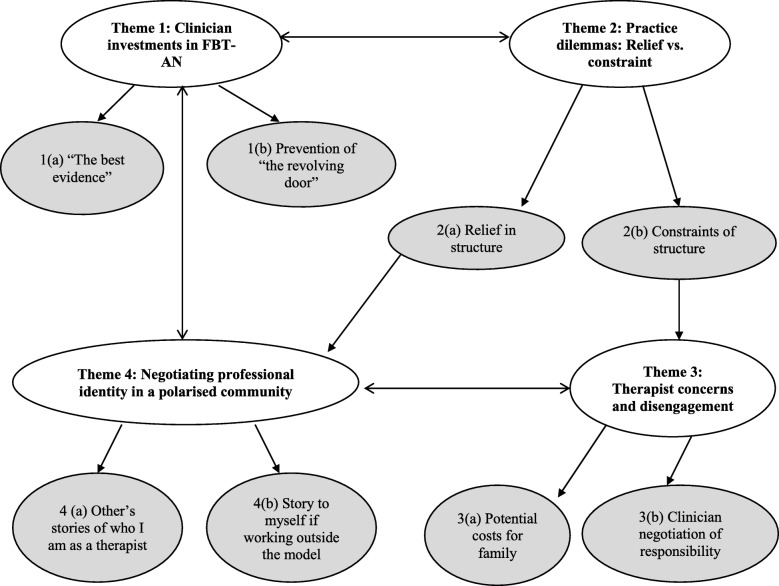
Thematic map of therapist engagement with FBT-AN

Interview data was constructed into four themes, each with two subthemes, that highlighted clinician investments in the FBT-AN intervention, therapeutic dilemmas and professional identity negotiations in working within and outside FBT-AN. Figure 1 depicts the themes and associations generated in the analysis of interview data.

### Theme 1: Clinician investments in FBT-AN

The therapists rationalised and justified their therapeutic implementation of FBT-AN by taking up the position that it is EBP and a window into early intervention in the prevention of AN running a chronic course.

#### The “best evidence”

All of the currently practising FBT-AN therapists were invested in the model and justified this positioning through a scientific discourse that elevated the knowledge of research evidence whereby EBP became reduced to the application of manualised ESTs.

##### Extracts 1

***Anja:*** *I guess what matters to me is feeling that people get […] best evidence sort of care. […] that I don’t just go with my gut feeling or […] on a personal perspective, but more from a professional perspective.*

***Josh:*** *I see myself as a therapist who deliberately chooses and adheres to evidence-based treatments, then I go and vary an evidence-based treatment [...] maybe I’m falling into the kind of hubris [...] It causes what we’ll call cognitive dissonance and some doubt about whether […] I am indeed an evidence-based therapist or not, because that is part of my identity.*

***Francesca:*** *I do strongly believe in evidence-based practice. Eating disorders are very complex illnesses and I don’t profess to know any better than the next person.*

Within these stretches of text, these clinicians’ identities were invested in being “evidence-based” practitioners. These therapists’ positioning on FBT-AN was built on a scientific repertoire [40] that constructed the research evidence as fact, and the implementation of FBT-AN was understood as synonymous with EBP. Nevertheless, the certainty of this position was also implicitly questioned through use of qualifiers such as “sort of” (Anja).

These therapists expressed concern not to elevate their clinical expertise over the model. Within some accounts research evidence was assumed to be “good” knowledge, thereby marginalising therapists’ expertise as a lesser knowledge (“personal perspective”) that, if relied on too heavily, was an unacceptable elevation of a therapist's expertise (“hubris”). Implicit in these dilemmas were these therapists’ commitment to accountability in their work as practitioners who provide effective treatment interventions to adolescents and their families.

#### Prevention of “the revolving door”

Six of the nine therapists derived a sense of meaning and purpose in their work through being part of the broader project of preventing “the revolving door” (Francesca) of AN.

##### Extracts 2

*Francesca: I felt extremely disheartened by the revolving door […] my sense is like there has to be some other way. So, what it has meant for me over the years, I guess, is something about feeling more effective as a clinician.*

*Glenda: I don’t have to bang my head against a wall for six years. So, I suppose it meant to me that I was part of a new edge of treatment […] that I was joining.*

*Marmot: I’ve got school-aged girls, and so it means a lot to me, personally […] girls having a strong sense of themselves, and not being railroaded into viewing themselves in a very narrow way [...].*

These therapists’ identities were supported in being part of a community that stood against adolescent AN running a chronic course. Working as an FBT-AN therapist contributed to a sense of oneself as engaging in effective therapeutic practice (Francesca), aligning oneself with cutting edge therapeutic practices (Glenda) and standing with young women in the development of durable and diverse identities (Marmot).

#### Theme 2: Practice dilemmas: relief vs. constraint by the structured intervention

The therapists were both comforted and constrained by the structured manualised approach that, in the earlier phases of therapy, strategically narrowed therapeutic conversations to prioritise focus on the adolescent’s eating and weight gain.

#### Relief in a structured approach to adolescent AN

Seven of the nine therapists experienced relief in, and justified, the structured approach of the FBT-AN manual.

##### Extracts 3

*Anja: I find really helpful that it’s a very clear structured approach […] [Giving] families […] [a] focus on which is re-feeding rather than you know, going into the depths of why, what and how.*

*Paige: My initial reaction to it [FBT-AN] was fantastic, like, this is something where we get lots of containment […] I felt confident that I could manage all these different things that were going to come up.*

*Francesca: Working with families initially wasn’t (pause) I wouldn’t say it was overwhelming, but it was like just how do you manage that? Many voices and personalities in the room? I think having the structure of something like FBT gives you a sense of confidence in the room.*

Anja understood the structured approach of the FBT-AN script as a way of shifting the focus from questions for which there may not be a clear answer. Paige and Francesca found that, particularly when they were inexperienced therapists, the structure of FBT-AN provided containment of a parallel process of anxiety between the adolescent, their family and themselves as therapists.

#### Constraints of a structured approach to adolescent AN

Eight of the nine therapists recounted feeling constrained by the structured approach of FBT-AN and both implicitly and explicitly questioned rigid adherence to the model.

##### EXTRACTS 4a

*Paige: You’re being really directive […] And it feels like actually harming […] but I know that’s what’s going to get the patient well.*

*Glenda: Everybody knows exactly where they are at any one time. Now of course that also had downsides too. Because you were sacrificing important conversations for staying on track.*

*Josh: The treatment, necessarily, comes at the expense of talking about the adolescent’s emotional state and individual needs, […] and that’s what makes it work, quite frankly.*

*Anja: Ideally, I’d like them to be engaged with me […] but they hate me, that’s alright. As long as they eat.*

These therapists expressed concerns about FBT-AN, particularly in the earlier phases, being “really directive” (Paige), and that this ultimately led to “sacrificing important conversations” (Glenda) such as the “adolescent’s emotional state” (Josh) or undermining an “ideal” therapeutic engagement (Anja). These therapists were caught in an ideological dilemma that was generated in contexts where the FBT-AN script was elevated over their own clinical expertise and their clients’ preferences (“they hate me”, Anja). These negotiations were built on a moral discourse that adopted a consequentialist ethical framework [41] whereby these therapists were able to justify their temporary neglect of the adolescent’s “emotional state and individual needs” (Josh) to keep “on track” (Glenda) in the service of saving lives.

##### EXTRACT 4b

*Francesca: I think having a model that you could kind of rest into reasonably confidently, I think that gives you confidence as a clinician as well. Obviously, as you become more experienced you can become more flexible with use of the model.*

One of the therapists did not experience FBT-AN as constraining in her work but rather as a model to “rest into” (Francesca, extract 4b) with augmentations assumed (“obviously”) to arise out of a therapist’s growing expertise and confidence.

##### Extracts 5

*Marmot: She says to her parents when she’s not feeling well [...] “I’m not learning anything about looking after myself, because you tell me what to do with what I eat and exercise.” And you know, she’s got a point; but the problem is, if they didn’t, she’d just be back in hospital.*

*Paige: I think the way I’ve been trained has been very rigid, it’s almost like ‘give a man a fish, he’ll eat for a day, teach a man to fish and he can feed himself’. I almost feel like I’ve been given fish with this model.*

Within these stretches of text, there existed a parallel process between adolescents and the clinicians who treat them where they were both told what to do through FBT-AN (“given a fish”) that had the effect of impeding the development of their own sense of competence around tailored formulation-based treatment (“teach a man to fish”).

Overall, the status of the FBT-AN manual as “good” or a more valid form of knowledge risked disempowering adolescents and the clinicians who treat them through the marginalisation of their own knowledge and expertise in AN interventions and recovery.

### Theme 3: Therapist concerns and disengagement from FBT-AN

FBT-AN became increasingly dilemmatic for some therapists in contexts where the adolescent did not progress to weight restoration. At these points in their narratives, they negotiated questions of responsibility and blame, and their role as therapist became increasingly burdensome.

#### Therapists’ concerns for the potential costs for families

Seven of the nine therapists expressed concern about the expectations and lack of resources for parents within this therapeutic intervention.

##### Extracts 6

*Sebastian: We had [hospital] staff […] highly knowledgeable, very specialized […] eight hours work with these patients and they’re drained […] I appreciate that one of the tenants of Maudsley is that parents have the ability to re-feed their child […] But what we’re asking them to do is outside of the realms of normal parenting.*

*Gloria: The families where it worked either very well or well enough, they gave up huge income to do it […] This is what we need to do to save our daughter’s life.*

*Paige: Probably we underestimate how difficult it is for parents to treat their young person.*

These clinicians were troubled by the core FBT-AN premise that it is reasonable to ask parents to take on primary responsibility for refeeding their child and the emotional and material effects of this. Implicit in these clinicians’ arguments is the extent by which the FBT-AN model risks under-acknowledging and underestimating emotional and material cost on parents who are asked to take responsibility for re-feeding their child in the early phases.

##### Extract 7

*Francesca: I can’t say that putting parents in charge is unhelpful to kids […] I think it is very challenging, but I think it’s extremely helpful. […] I think that the families who don’t go well are the families in which mum and dad struggle to take charge.*

In contrast, Francesca positioned the task asked of parents as “challenging” but necessary. Drawing on the discourse of structural family therapy [42], she argued the families who do not do well with the intervention are those where the parents struggle to “take charge” and that “this doesn’t mean they’re to blame, it means this treatment may not be for them”.^2^ A number of questions following on from this position, including how do therapists discern that FBT-AN intervention may not be appropriate for an individual and their family and how are alternative treatment options navigated?

#### Clinician negotiation of responsibility

In contexts where FBT-AN did not work, the question of responsibility was raised by eight of the nine therapists..

##### Extracts 8

*Francesca: I’m not responsible for change, like if the kid doesn’t get well or a family doesn’t respond to the treatment, and I need to remember that it’s not going to work for everybody.*

*Anja: I guess that’s just a value of therapy. Therapies never work out how we want them to.*

*Paige: The feedback that I’ve been given is keep going within the model but just always try harder […] it undermines my confidence because I think I’m not doing it right or not doing it well enough.*

*Josh: Whenever any client isn’t doing well […] I go through quite a deliberate, conscious process of thinking […] That’s a conscious self-doubting, self-examination, but I’m in favour of that.*

In order to sustain themselves in the context of therapeutic challenges, Francesca and Anja negotiated the causal discourse of responsibility and blame through reminding themselves that outcomes are complex and collaborative. On the other hand, feedback from her supervisor to “keep going within the model” and “try harder” eroded a sense of professional competence for Paige. Josh preserved a sense of professional identity when a client was “not doing well” through positioning self-doubt as a form of self-examination that was aligned with his professional values.

The theme of responsibility also arose when the therapists narrated some of the more troubled experiences that contributed to their decision to ultimately cease working as FBT-AN therapists.

##### Extracts 9

*Gloria: It’s the therapist’s job to sit with that distress and help them sit with their distress and get their thinking back so that they can keep their child alive and help them get well. That is a lot to hold […].*

*Glenda: […] I wanted so badly to be a good Maudsley practitioner and I’d had good experiences* [with adolescents and their families with FBT-AN] *and I wanted to follow the encouragements my supervisors were giving me, but in my heart I thought I don’t think this is right. […] The manual did not then, I don’t know if it has changed, address any sort of concept of window of tolerance of the young person. [...] In my FBT training and ongoing supervision I was not taught or invited to be aware of when the distress may be so impactful it becomes a new set of problems –* i.e. *trauma. Looking back […]I would have challenged my supervisor and stated that in my opinion there is a limit to the distress that I would be willing to normalise within FBT. […] I have left behind the dogmatic culture of FBT, which I believe cloaked my capacity to act on what felt uneasy because it was not adhering to the model. I am somewhat haunted by the sense the model was used to ‘justify the unjustifiable’.*^3^

These therapists recounted their struggles to “sit with” and “hold” (Gloria) the distress of an adolescent and their family in the FBT-AN treatment. An experience that contributed to Glenda’s decision to cease practising as a FBT-AN therapist was the implicit risk of an intervention that heavily relies on parents to engage in home-based refeeding of their child in the early stages of treatment with less focus or consideration of the level of distress that this may invoke for the adolescent and within the family system. This approach, where the ends justifies the means, reduced Glenda’s personal agency as an early career FBT clinician in the field and she experienced a parallel process [43] of distress that was unable to be contained by the ongoing strict adherence to the model in supervision.

### Theme 4: Negotiating professional identity in the context of a polarised community

All the therapists expressed reticence in working outside the framework of FBT-AN. How they ascribed meaning to working with adolescents and their families outside FBT-AN was shaped by the stories they believed others might tell about them as a therapist and the stories they told themselves to preserve an identity sourced in their values.

#### Others’ stories of who I am as a therapist

The therapists’ stories of self, when working outside FBT-AN, were shaped by the broader professional context.

##### Extracts 10

*Josh: My supervisor says […] I should work harder to make sure that everybody’s there, all the time, in fact, they say I’m not really doing Maudsley, if the siblings aren’t in the room.*

*Sebastian: One of the comments that stands out for me is one of the trainers saying if Maudsley’s not working then the clinician’s not doing their job.*

*Joy: So, I feel like I just don’t have the capacity or the interest really to be carrying […] “She’s someone who works with adolescents in a non-evidenced based way.”*

These therapists talked about the power of the supervisors’ gaze overseeing and evaluating their work, particularly when departing from FBT-AN. Josh described an obligation to adhere to the model as his supervisor construed any deviation, even implicitly, as not working hard enough. Sebastian was troubled by his supervisor’s assumption that if FBT-AN was not working then the therapist was to blame. Joy decided she did not want to carry other’s label of her as a therapist who works “in a non-evidenced based way” and the material effects of this were her decision to cease seeing adolescent’s and their families.

##### Extracts 11

*Joy: The Boys’ Club, I just feel like there’s quite a lot of bullying, like not in a really - in a covert sort of way […] Some of the phrases that they use, which when I’ve had supervision, by them; […] “You’ve got to pull them in before you punch them.”*

*Glenda: I know of people who have been challenged by Maudsley cultists almost. They say, “well we want to know what you’re doing seeing adolescents if you’re not going to offer this.”*

These therapists, who had ceased using FBT-AN, expressed concern about rigid and strict adherence to the model. Joy exaggerated these concerns to the level of “Maudsley cultist almost” to highlight the investments in the rigid application of the model within the broader context of a gendered profession (“the boys club”). These terms capture the implicit effects of extreme fidelity to FBT-AN that work to marginalise and de-legitimise those who offer treatments through other frameworks of care. The real effects of these power dynamics between therapists in the field of ED’s led to three of the therapists in this research to not only cease working within the model but to also abandoning work with adolescent AN. For these therapists, the fear and risks of not working within the model and being recruited into the identity of non-evidence-based practitioners were too great.

Five of the six therapists who continued their work as FBT-AN practitioners at the time of the interview remarked on the effects of a split treatment community.

##### Extracts 12

*Josh: The ones that are rigid [...] are the ones that aren’t educated, and don’t use it [FBT-AN] at all. I’ve got a couple of colleagues, psychiatrists that will even tell their patients, “Oh, don’t do Maudsley, that’s just a fad.”*

*Francesca: My job becomes infinitely harder because it’s very hard to hold a bottom line in treatment with a young patient when there is no sort of medical review or support in place […] so I would like more support, more awareness dissemination.*

Josh positioned the divide between those who are “educated” and practise FBT-AN, and those who are “rigid” and firmly oppose it. Being part of a community where some professionals were resistant to the potential benefits of FBT-AN also was experienced as presenting risks to client safety. Francesca’s commitment to “hold a bottom line in treatment” in standing for the adolescent’s medical safety in a community that was not informed of FBT-AN was experienced as isolating and unsupported.

#### Story to myself: who I am as a therapist if I work outside the model?

The “choice” to work outside of the script of FBT-AN was dilemmatic for the majority of the therapists as they engaged in identity negotiations and justifications as to why.

##### Extracts 13

*Sebastian: I think you’ve got to sort of draw a line in the sand and say okay well do we just keep doing what we’re doing, knowing that the weight’s not going on and the parents are getting stressed and the relationships are disintegrating, or do we start looking at other options.*

*Joy: I feel it’s a bit disrespectful to the clients…. to build rapport before you hit them with something that’s going to shatter them. […] I feel uncomfortable with the way that they [FBT-AN trainers/supervisors] talk […] just disrespectful, actually.*

Sebastian argued that his choice to work within the model involved the adolescent and their family. Drawing on the metaphor of “a line in the sand”, he highlighted the point at which a therapist is faced with giving the adolescent and their family more of the same intervention or switching direction to draw on other therapeutic interventions. Joy’s concerns with the model were centred upon a values clash where she no longer wanted to participate in therapeutic practices that she experienced at times as disrespectful and holding the potential to “shatter” the adolescent and their family.

All the therapists were also faced with what it meant to them if they were to draw on other therapeutic interventions when working with adolescents who experience AN and their families.

##### Extracts 14

*Josh: The families and the kids find it (pause), they kind of go, “That’s frustrating!”, and it’s one of the things that leads to drop out rates. They say, “You’re not talking about what’s really going on”.*

*Marmot: I would find it very difficult to just follow a manual, because I would feel that I was compromising on my clinical ability – and my values. I was just kind of forcing people through something, when in my head, my clinical experience is going, “This isn’t right; this isn’t going to be as effective if I do it in this way.” [...] I don’t know if that’s actually true, but that’s my sense.*

*Joy: I mean I feel torn because there’s a part of me that would like to see a bit more capacity for people to work freely according to their clinical judgment, but then I see that there’s a lot of danger in that as well.*

Implicit in each of these stretches of text were these therapists’ values including authenticity (“talking about what’s really going on”; Josh), accountability (not “forcing people through something” that “isn’t going to be as effective”; Marmot) and flexibility (Joy). These therapists’ professional identities were sourced in their values yet also troubled by a fear that drawing on their clinical expertise risked “danger” (Joy).

## Discussion

This study highlights some of the commitments and professional identity negotiations of therapists who practise or have practised FBT-AN. These therapists were committed to adhering to FBT-AN and frequently experienced themselves as engaging in therapeutic processes that were effective and created positive change for adolescents and their families. These experiences contributed to their identity investments as evidence-based practitioners. On the other hand, eight of the nine clinicians highlighted a range of difficulties in negotiating their professional identities and sustaining themselves in this work that became increasingly burdensome in contexts where they took up personal responsibility for the adolescent’s eating and behaviour change. One clinician derived a durable and relatively untroubled sense of identity through her practice as a FBT-AN therapist. Three of the practitioners had ceased working not only with the FBT-AN model but also with adolescents who experience AN.

The script of FBT-AN was experienced as a relief and justified through the prioritisation of the adolescent’s medical safety and in the prevention of AN running a chronic course. However, these commitments became morally dilemmatic in contexts that included when the intervention did not work, witnessing the burden placed on parents in taking on the role of refeeding their child, the adolescents’ loss of voice and marginalisation of their emotional distress to focus on eating and weight restoration in the earlier phases of treatment, and when adolescents and families dropped out of treatment.

These therapists’ identities were invested in being “evidence-based practitioners”, which was often self-evidenced by adherence to the FBT-AN manual [2, 3]. This knowledge was positioned as “good” knowledge [44] through a scientific repertoire that elevated research evidence over the therapists’ clinical knowledge and expertise. The power of the scientific discourse was evident by the swift assumption made by clinicians that strict fidelity to the manual was a required component of EBP. The uptake of this discourse was evident where clinicians were troubled by departures from the FBT-AN script and in the marginalisation of their own knowledge and expertise of what intervention/s might work better at a particular time in treatment for an individual adolescent and their family.

Previous research has found that therapists do not fully ascribe to FBT-AN for a range of pragmatic and ideological reasons, including the belief that “one size does not fit all” ([26], p., 182). What is less known is how their negotiations and actions to modify this intervention shape their sense of professional identity. These therapists ascribed a range of meanings to deviations from the model when relying on their clinical intuition, including feeling burdened by doubt, and fear they were biased and inflating their self-importance. One clinician was untroubled by drawing on her own expertise to augment the FBT-AN model and argued that this may at times be of greater therapeutic benefit to the adolescent and their family. All the therapists, however, were reticent to draw on their clinical expertise to question to basic tenets of the model or to transform (rather than merely augment) its structure when working with adolescent AN.

This research also highlights the divisive effects of a polarised community of practitioners where therapists were recruited into “choosing” between “us and them” within different contexts. Those who no longer practised FBT-AN experienced themselves as outsiders to the ED community. This marginalisation was experienced by some as an act of power through the assumption that FBT-AN was the only means to engage with EBP, thereby disqualifying those clincians who drew from different therapeutic modalities for adolescent AN. On the other hand, those who practised FBT-AN experienced themselves as misunderstood and unsupported by the broader community of health professionals, thereby posing a risk to collaborative care.

These “us and them” debates in the clinicians’ narratives paralleled those within the broader ED community, such as those who argue for the effectiveness of the intervention [45] and those who argue that FBT-AN is “overvalued” ([23]: p. 264) and that AN treatments should be formulated and treated by a more complex reasoning process than by prescribing a single manualised EST. These debates also highlight the issues of power and masculinity in the neoliberal distribution of EST’s as a “product”, rather than promoting a process that embodies the original conceptualisations of EBP that draw on clinician expertise to tailor clinical interventions to the needs, preferences and values of the client [21].

### Implications

This research highlights that the polarisation of the ED community through the uptake of the dominant discourse of EBP risks therapists being isolated, undermined, confused or scrutinized about their treatment decisions, whether they adhere to FBT-AN, implement alternative therapeutic modalities or make augmentations to the model. This research makes transparent this research-practice gap and considers how the subjective experiences of clinicians may inform future practice.

These clinician narratives highlight some key areas to bridge this research-practice gap that include:

1. The establishment of more comprehensive guidelines and ongoing assessment of “the appropriateness and efficacy of FBT(-AN)” ([46], p., 298) in therapeutic contexts including for example, when:
  - The adolescent’s emotional distress (including in their eating and weight restoration) is too great and the intervention risks being traumatising;
  - The burden on a family is too great and what might be the next steps in the care of the adolescent and their family; and
  - There exists a risk of child emotional abuse and family violence, thus highlighting the need for therapists who practice FBT-AN to have comprehensive training in responding to child emotional abuse and family violence [46].
2. Drawing on therapist expertise to both inform clinical practice guidelines [25] and future research into how to effectively tailor manualised treatments to the adolescent and family needs and preferences through reflective practice in supervision [43].
3. Addressing the pressing need for the development and evaluation of more diverse treatment interventions for adolescent AN, including those that have scope to be flexibly tailored by clinicians to the needs and preferences of the adolescent and their family. These treatments need to prioritise approaches that give the adolescent a voice and scope for choice [47] alongside treatment “non-negotiables” such as weight gain [48].
4. Further development of supervision methods for FBT-AN that cultivate an awareness of some of the implicit messages that clinicians may take up when the focus is on strict fidelity to the model and where any therapeutic departures are scrutinised and interrogated and how this may disempower therapists and risk burnout.
5. Researching ways reflective practice in supervision [43] might empower ED therapists to strengthen their connection with their preferred identities, sustain themselves in clinical practice, and to continue to develop expertise in the discernment of ways to effectively tailor therapeutic interventions including through prioritising the voice of the adolescent and their families [49].

While there is a proliferation of studies exploring augmentations to FBT-AN, this does not address the gap between therapist adherence to the manual and when and how to draw on their clinical wisdom. These augmentations tend to accommodate other interventions onto the model and are reluctant to corrupt the core principles of FBT-AN [50] that include allocation of responsibility of re-feeding the adolescent to parents in the early phases of treatment. Furthermore there continues to be a reluctance to engage in transformative models of care in adolescent AN and a paucity of research into such other treatment paradigms [8].

### Scope of the study

This research is an in-depth analysis of the views of nine Australian clinicians. It is possible that the research advertisement for recruitment of participants that put forward an interest to explore ways to “improve” FBT-AN shaped the study participants such that those who were troubled by the model were more likely to participate. Nevertheless, there was a broad range of experiences and positionings articulated by these nine practitioners. It would be valuable to explore whether clinician views differ, particularly in the context of diverse cultures, countries and healthcare systems.

While the interview questions were intended to investigate both the helpful and unhelpful aspects of FBT-AN, it is possible that the telling or analysis may not have equally captured both, for example due to a retrospective bias on accounts that have more emotional valence. As well as this, as the interviewees themselves could not be anonymous to the interviewers, it is possible this may have limited their disclosure in an effort to protect aspects of their professional identity. Lastly, it is worth noting this paper has drawn on Sackett et al.’s model [21] of EBP, which rather than elevating research evidence over clinician and client expertise construes it as an interplay between these different forms of knowledge. This positioning may diverge from those who argue that “scientific evidence must be accorded priority” ([51]: p.886) when there is doubt in clinical decision-making.

## Conclusions

This research provides a unique insight into the experiences of therapists who practise FBT-AN, and the meaning making processes that they negotiate to sustain themselves in their work. While FBT-AN is an effective treatment for many adolescents, greater focus needs to be accorded to the role of clinician expertise in the tailoring of therapeutic interventions to client preferences rather than the strict application one EST, which for the majority of these therapists was dilemmatic. Through developing a two-way relationship between research and practice, there exists scope to expand therapeutic interventions for adolescent AN that embody the intended meaning of EBP that is, to provide clinicians with scope and a more comprehensive map to tailor the best possible treatment interventions to the needs, preferences and values of the individual adolescent/family [21].

## Additional files

Additional file 1: Participant demographic and professional history questionnaire. (DOCX 23 kb)

Additional file 2: Semi-structured interview questions for participants. (DOCX 22 kb)

## Data Availability

A full thematic map including quotes may be requested from the corresponding author upon reasonable request.

## Abbreviations

AN: Anorexia Nervosa
EBP: Evidence-Based Practice
ED: Eating Disorder
EST: Empirically Supported Therapy
FBT-AN: Maudsley and Family-Based Therapy
RCT: Randomized Controlled Trial

## Footnotes

1 Although Maudsley Family Therapy (Family therapy for eating disorders originating from the Maudsley London Hospital under the Eisler group) (Russell, 1987); is not exclusively synonymous with Family-Based Therapy for Anorexia Nervosa (the manualised treatment based on the Maudsley treatment by Lock and Le Grange in the USA), this article will refer to these interventions with the term FBT-AN, acknowledging that some of the therapists who had worked for a longer period of time with the model would have been trained in either or both of Maudsley Family Therapy and FBT interventions.

2 This quote was ascertained from a member-check correspondence.

3 This quote was ascertained from a member-check correspondence
