# Supplementary material for: “As long as they eat”? Therapist experiences, dilemmas and identity negotiations of Maudsley and family-based therapy for anorexia nervosa
Source: J Eat Disord. 2019 Aug 1;7:26. doi: 10.1186/s40337-019-0255-1 (PMC6670233; doi:10.1186/s40337-019-0255-1)
Supplement: Supplementary file 1 — Participant demographic and professional history questionnaire. (DOCX 23 kb) [file 40337_2019_255_MOESM1_ESM.docx]

Appendix A

**Demographic and Professional History Questionnaire**

*Project Title: Experiences of health care professionals in treating adolescent AN with the Maudsley family-based therapy approach*

Please answer the following questions to your best knowledge.

**General Information**

Participants name:

Address:

Contact number:

Date of Birth:

Age:

**Professional History**

1. What is your current discipline?
2. How many years have you been working in this discipline?      years
3. How many years of experience have you had working with individuals with EDs?      years
4. How many individuals with EDs have you seen (approximately)?      individuals
5. Have you been trained in Maudsley FBT?

Yes

No

1. If yes, what type of training? (Please select as many as applies to you)

Reading the manual

Reading sections of the manual

Workshop (Equal to or less than 2 days)

Workshop (More than 2 days)

Training seminar

Please specify how long in days:       days

University/work training

Please specify how long in days:       days

Other (Please specify what type and length of training)

1. Have you participated in supervision for Maudsley FBT?

Yes

No

1. If yes, how many hours?      hours
2. How many years have you been practicing Maudsley FBT?      years

Appendix A continued

1. Have you participated in any generic family based therapy training?

Yes

No

1. If so, what type and for how long?
2. Do you currently use any other therapy models for individuals with EDs?

Yes

No

1. If yes, what types?
2. If yes, have you been trained and for how long?

**Past Professional History**

1. What (if any) were your past disciplines?
2. What (if any) past training do you have, or have you used with individuals with EDs?
3. If you have used other training techniques or therapy models for individuals with EDs (in the past), what type and for how long did you utilise it/them?

**The following questions are about various aspects of Maudsley FBT:**

1. Do you weigh the clients each session?

Yes

No

1. If not, why?
2. How do you discern when to move from Phase I to Phase II of Maudsley FBT?
3. Do you agree on the position in Maudsley FBT that “all other issues that the family has had to postpone related to eating is to be processed in Phase II”?

Yes

No

1. How do you experience this in therapy?
2. Do you find the transitional phase a smooth process?

Yes

No

Appendix A continued

1. Can it be premature at times?

Yes

No

1. How do you experience this part of treatment?
2. Do you always conduct a family meal with the aim of coaching the parents to take control of their child’s eating?

Yes

No

1. Are they helpful for the effectiveness of the therapy?

Yes

No

Both (please give further information)

1. In your experience, do you find parents generally share the same strategies or approach when working with their adolescent child/children?

Yes

No

1. Does this affect the effectiveness of Maudsley FBT with the client?

Yes

No

Other (please give further information)

1. Do you externalise AN with your client?

Yes

No

Other (please give further information)

1. Do you feel supported using the Maudsley FBT manual/approach to therapy?

Yes

No

1. If not, how did/does this feel for you?
2. In your experience, can you work within a Maudsley FBT paradigm if you do not feel supported within the eating disorder community (including inpatient and day patient programs)?

Yes

No

Other (please give further information)

Appendix A continued

1. In your experience is Maudsley FBT more beneficial for outpatient or specialist inpatient/day programs?

Outpatient

Specialist inpatient and day program

Both

None

1. Are there any aspects of the Maudsley FBT manual/approach to therapy that you find really helpful?
2. Are there any aspects of the Maudsley FBT manual/approach to therapy that you find not helpful, that you avoid, or would like to change?
3. Finally, what was it like to answer those questions?
