# Supplementary material for: “As long as they eat”? Therapist experiences, dilemmas and identity negotiations of Maudsley and family-based therapy for anorexia nervosa
Source: J Eat Disord. 2019 Aug 1;7:26. doi: 10.1186/s40337-019-0255-1 (PMC6670233; doi:10.1186/s40337-019-0255-1)
Supplement: Supplementary file 2 — Semi-structured interview questions for participants. (DOCX 22 kb) [file 40337_2019_255_MOESM2_ESM.docx]

Appendix B

**INTERVIEW QUESTIONS FOR HEALTHCARE PROVIDERS**

**A selection of questions will be used with each participant; questions will scaffold between**

- **Experience (e.g. Can you tell me about …?)**
- **Meaning (What does … mean to you?)**
- **Identity (e.g. How does … have you seeing yourself as a person)**
- **Positioning on experience/identity conclusion (e.g. is this OK for you or not? Why?)**

**A) 1.** What does working with people who experience AN and their families mean to you as a health professional?

1. How has your professional life changed with working with people who experience AN and their families? What have you got in touch with yourself as a health professional?
2. Do you see many adolescents and their families for treatment for AN? Has this changed over time? Why or why not?

**B) EXPERIENCES OF MAUDSLEY FAMILY-BASED THERAPY (Maudsley FBT)**

1. What have been your experiences of working with adolescents and their families within the therapeutic paradigm of the Maudsley FBT as a health professional?
2. What have you found most helpful about FBT for individuals and the families you have worked with? Why?
3. What was least helpful about FBT for individuals and their families? Why?
4. Do you presently work within a Maudsley FBT framework with adolescent anorexia?
5. If you are working with an adolescent and their family with M-FBT and things are going well, how does this affect you? What are the thoughts going through your mind? How does this affect your view of yourself as a clinician? Helpful or not? Why or why not?
6. If you are working with an adolescent and their family with M-FBT and things are not going well, how does this affect you? What are the thoughts going through your mind? How does this affect your view of yourself as a clinician? Helpful or not? Why or why not?

**If STILL WORKING IN M-FBT PARADIGM**

1. What have you found to be the most helpful components of this intervention?
2. Do you have any de-identified stories of families for whom Maudsley FBT was helpful?
3. What sustains you in this work?
4. How does working within this framework affect how you see yourself as a health professional?
5. What do you do if an adolescent and their family is struggling with M-FBT? How do you discern this and what steps do you take in therapy? How does this have you seeing yourself as a therapist?
6. Do you find sometimes that you draw on other therapeutic paradigms for adolescents and their families? How does this affect how you see yourself as a therapist? Is this helpful or not? Why or why not? What might you be prioritizing/valuing when you make such decisions to draw on other therapeutic interventions with some adolescents and their families?
7. When using the M-FBT therapeutic framework, did you use techniques that are not recommended either explicitly or by their absence in the manual? (e.g., individual therapy; mindfulness techniques). If so, what did you find useful? If so, what did you find not so useful? How did drawing on these other orientations affect how you see yourself as a therapist? Helpful or not? Why or why not?

**IF CEASED WORKINGIN M-FBT PARADIGM**

1. If no, what were some of the reasons that you chose to stop working within this therapeutic framework?
2. Did or do you have any fears or concerns about either continuing or discontinuing working as a Maudsley family based therapist?
3. How ceasing working as a Maudsley family based therapist affect how you see yourself as a health professional?
4. In ceasing working as a Maudsley FBT, do you continue to work with adolescent AN and if so, what treatment approaches do you draw upon in this work? How does working within these therapeutic paradigms affect how you see yourself as a therapist?

**The following questions will be asked about particular aspects of Maudsley FBT if participants do not cover these areas from the above open lines of questioning.**

**Weight restoration/phases of treatment**

1. Do you weigh your clients each session? If not, why?
2. How do you discern when to move from Phase I to phase II of Maudsley FBT?
3. What are your thoughts on the position in M-FBT that “all other issues that the family has had to postpone related to eating is to be processed in Phase II”? How do you experience this in therapy? How does this feel for you? How does postponing other matters affect some members of the family or some adolescents?
4. How do you experience the phase of transition to adolescent independence? When do you discern that an adolescent is ready for this transition? How do you find a family navigates their way through this transition? How do you experience this part of treatment? (Probe: Do you find the ‘transitional phase’ a smooth process? Can it be premature at times? What are your experiences with this phase and if any lapses?)

**Family meal and parental instruction at family meals**

1. Do you always conduct a family meal with the aim of coaching the parents to take control of their child’s eating? What stands out for you from these meetings? Are they helpful or not for the effectiveness of the therapy? Or both? Why?
2. If so, what is this session like for you as a therapist?
3. How do you experience asking an adolescent who experiences AN to “*resist the parents’ attempts at getting her to eat during the family meal*” in order not to “*miss the therapeutic opportunity to give the parents a direct experience of empowerment*” feel for you as a therapist?
4. How do you work with parental expertise? Do you feel you have enough of a scope on the parents to step back and watch ‘parental expertise’?
5. What effects have you seen when parents are asked to be ‘relentless’ with their child’s AN? Can you tell me how you experience this?

**Externalisation of anorexia as an illness**

1. What are your experiences of externalisation of AN as an illness?
2. How do you find adolescents’ and their families experience externalising their experiences as anorexia?

**Parental Unity**

1. How do you experience working where you are promoting parental unity?
2. What happens if the parents are not united? How important, do you think, is this to M-FBT?
3. In your experience, how often were parents united? Was this consistent throughout treatment?
4. Do you ever need to do additional work beyond M-FBT with the parents to help create or maintain a ‘united front’? How did you feel about this? How did this affect how you saw yourself as a therapist?

**Sibling support**

1. How do you recruit sibling support and what’s your experience of this as part of M-FBT? Do you follow the manual in this, or not? And why? Or why not?

**Team support**

1. Do you feel supported using the M-FBT manual/approach to therapy? If not, how did/does this feel for you?
2. The manual states to work in a ‘team’ setting or with ‘support structures’ – do you feel like you have these working with your clients with AN?
3. Can you work within a M-FBT paradigm if you do not feel supported within the ED community (including inpatient and day patient programs)?
4. What does it mean for you when you need to refer an adolescent and their family to a specialist ED inpatient/day program?
5. In your experience is M-FBT more beneficial for outpatient or specialist inpatient/day programs?
6. When M-FBT does not work for a client, what factor, in your experience, are involved? How does this make you feel?

**Questions to all participants regardless of whether continued or discontinued working as a Maudsley family based therapist.**

1. What do you want to not forget about yourself in relation to your work as a Maudsley family based therapist?
2. What advice might you give to a family who is about to commence Maudsley FBT?
3. What would you like more of in future treatments for anorexia? What would you like less of in future treatments for AN? What does this say to you about what matters for you as a health professional?
4. Do you have hopes for future treatment/therapy in terms of the sort of work you would like to engage in? What does this say to you about what you value as a health professional?
5. Has our conversation today been helpful or unhelpful or both? Why?
6. What has stood out for you from our conversation today?
7. What might be important for us not to forget as we analyse the data from this interview?
